# Two common structural motifs for TCR recognition by staphylococcal enterotoxins

**DOI:** 10.1038/srep25796

**Published:** 2016-05-16

**Authors:** Karin E. J. Rödström, Paulina Regenthal, Christopher Bahl, Alex Ford, David Baker, Karin Lindkvist-Petersson

**Affiliations:** 1 Department of Experimental Medical Science, Lund University, BMC C13, 22 184, Lund, Sweden; 2 Department of Biochemistry, University of Washington, and Howard Hughes Medical Institute, Seattle, Washington 98195, USA

## Abstract

Superantigens are toxins produced by *Staphylococcus aureus,* called staphylococcal enterotoxins (abbreviated SEA to SEU). They can cross-link the T cell receptor (TCR) and major histocompatibility complex class II, triggering a massive T cell activation and hence disease. Due to high stability and toxicity, superantigens are potential agents of bioterrorism. Hence, antagonists may not only be useful in the treatment of disease but also serve as countermeasures to biological warfare. Of particular interest are inhibitors against SEA and SEB. SEA is the main cause of food poisoning, while SEB is a common toxin manufactured as a biological weapon. Here, we present the crystal structures of SEA in complex with TCR and SEE in complex with the same TCR, complemented with computational alanine-scanning mutagenesis of SEA, SEB, SEC3, SEE, and SEH. We have identified two common areas that contribute to the general TCR binding for these superantigens. This paves the way for design of single antagonists directed towards multiple toxins.

Superantigens (SAgs) are immune stimulatory toxins secreted by bacteria, such as *Staphylococcus aureus*, which are capable of evoking an immune response of large proportions^1^. This ability stems from their capacity to cross-link the major histocompatibility complex (MHC) class II on the antigen-presenting cell with the T cell receptor (TCR) and activate large fractions of the T cell population^2^. SAgs are extremely potent and can induce a measurable activation of T cells (both CD4^+^ and CD8^+^) in the picogram concentration range resulting in production of cytokines such as tumor necrosis factor (TNF) α and β, and interleukin-2 (IL-2)^1^. This mitogenic effect commonly results in food poisoning, fever, and can lead to multi-organ failure and death. The advantage for *S.aureus* to harbor the genes for these toxins still remains unclear, but one hypothesis suggests that excessive T cell expansion causes immunosuppression^3^. Superantigen toxicity by inhalation is well established, and although exposure through this route is not a feature of *S. aureus* infection, this characteristic makes superantigens a candidate for use in biological warfare^4^. These threats underline the importance of developing superantigen antagonists.

*S. aureus* secretes multiple superantigens, called staphylococcal enterotoxins (abbreviated SEA to SEU). SEA is of particular interest due to its pronounced activity in humans, as it is able to activate human T cells a thousand-fold stronger than murine T cells^5^. Furthermore, SEA is the enterotoxin considered to be the main cause of food poisoning, probably due to its extraordinarily high resistance to proteolytic enzymes^6^. A comprehensive study of 359 outbreaks that occurred in the United Kingdom revealed that 79% of the *S. aureus* strains produced SEA. Moreover, SEA was also the most common enterotoxin recovered from food poisoning outbreaks in the US (77.8% of all outbreaks), followed by SED and SEB^6^.

SEB, on the other hand, is considered to be one of the most important toxin threats in bioterrorism^7^. Current scenarios of biological warfare and bioterror are likely to entail mixtures of multiple toxins. All staphylococcal enterotoxins are potent emetic agents, and most of the bacterial superantigens induce toxic shock syndrome^8^. This complexity demands the development of broad-spectrum countermeasures. Hence, an optimal superantigen blocker would simultaneously target several superantigens. This presents a difficult challenge due to the multiple modes of SAg interaction with TCR and MHC class II 9. When activating T cells, superantigens bind to either the variable α or β chain of TCR (TRAV or TRBV)^10^,^11^. The result of this interaction diversity is that different superantigens activate diverse subpopulations of T cells, and they do not bind in the exact same way to TCRs^12^,^13^. Still, most of the staphylococcal enterotoxins investigated so far use the same TCR-binding cleft to either bind to the TRAV or the TRBV domain of TCR^14^,^15^,^16^. When the staphylococcal enterotoxins bind to MHC class II, they either use the N-terminal domain, the C-terminal domain, or both domains^17^,^18^,^19^, hence this route poses additional challenges. Firstly, since the two MHC class II binding sites are distant from each other (on the opposite sides of the SAg molecule), it would be difficult to block both binding sites with a single antagonist, and secondly, some SAgs, such as SEA, can activate T cells by binding to other receptors, bypassing MHC altogether^20^. For instance, SEA has been shown to bind glycoprotein 130, using the MHC class II binding site^21^.

Taken together, there is an urgent need to gain insight into the mechanisms governing SAg-TCR interactions in order to design an optimal blocker of T cell activation by superantigens. The first step towards this is to identify which of the TCR-binding modes are common to several SAgs from the diverse interactions that mediate TRAV/TRBV specificity. In this work, we present the X-ray structures of SEA in complex with a human TCR, as well as SEE complexed with the same TCR. By combining these structures with the previously published SEB-TCR structure^14^, SEC3-TCR^15^ and SEH-TCR structure^16^, with *in silico* alanine scanning mutagenesis, we can make predictions about which residues are most important for protein complex formation. This is the first comprehensive analysis to tie all published data available, concerning TCR recognition by superantigens, together with computational analyses to identify a core set of conserved interactions present in SAg-TCR. This allows us to identify common structural elements likely to be useful for the design of broad spectrum SAg antagonists to abrogate SAg-TCR complex formation and neutralize the mitogenic activity of superantigens.

## Results

### Overall Structures of the SAg-TCR Complexes

**Figure 1:**
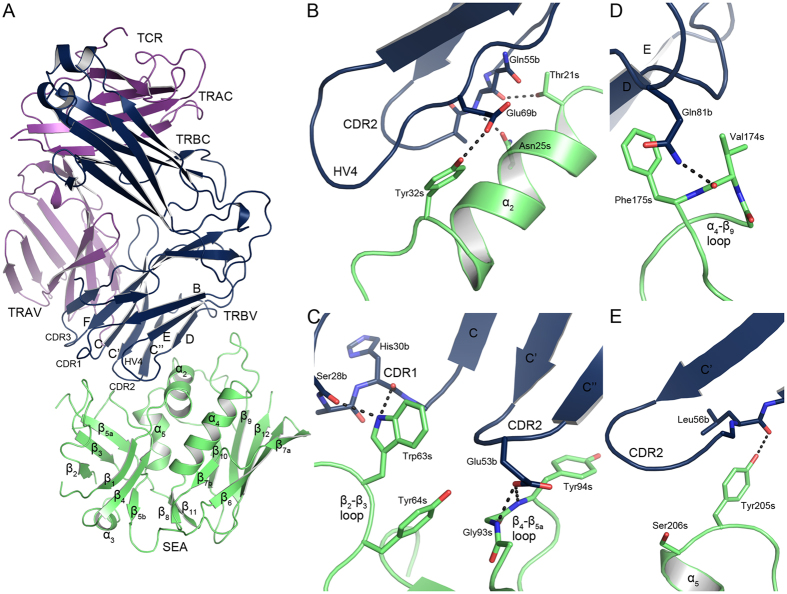
Structure of the SEA^F47A^-TCR complex. (**A**) Overall structure of SEA^F47A^-TCR, with SEA shown in green, and TCR in purple and blue for the α- and β-chain, respectively. (**B**) Close-up view of the SEA α_2_-helix and contacting residues in TCR, (**C**) the hydrophobic patch, consisting of the β_2_-β_3_ and β_4_-β_5a_ loops, (**D**) the α_4_-β_9_ loop, (**E**) and the upper part of the α_5_-helix. Hydrogen bonds are marked as black dotted lines and residues designated as “s” for SAg and “b” for TRBV7-9.

The SEA variant F47A, with a substitution in the MHC class II binding site not involved in TCR binding, was crystallized in complex with the human TCR variable domains TRAV22 and TRBV7-9 in space group P2_1_2_1_2, and the structure was determined to 3.1 Å resolution (Table 1). The structure was refined to R_work_ and R_free_ values of 25.71% and 29.08%, respectively and with 99.3% of all residues in preferred or allowed regions of the Ramachandran plot and 0.5% in the generously allowed region (Table 1). The remaining 0.2%, corresponding to one residue (Asn52 in TRBV7-9), is in the disallowed region. This asparagine, which is located at the apex of the CDR2 loop, is in the generously allowed region for the previously published structures of TRBV7-9 as well (4UDU and 4UDT)^22^. This residue is likely in a strained position, as it participates in a type II’ β-turn, and the backbone atom positions are not clearly defined in the electron density map in this region, which further complicates the refinement. TRAV22 and TRBV7-9 were isolated from two TCRs specific for HLA-A2 in complex either with a telomerase peptide (sequence ILAKFLHWL) or a survivin peptide (sequence ELTLGEFLKL), respectively. Attempts were made to crystallize the wild-type SEA-TCR complex, but this did not yield crystals of sufficient quality. The TCR has the same general fold as described previously for TCR^23^, with two constant domains (TRAC and TRBC) and two variable domains (TRAV and TRBV), almost exclusively consisting of β-sheets (Fig. 1A and Supplementary Fig. S2). We also observe the introduced disulfide bond between residues Cys160a and Cys170b in the constant domains^24^. SEA^F47A^ exhibits the conventional superantigen fold, as first described for SEB by Swaminathan *et al*.^25^. Briefly, SEA consists of two domains: an N-terminal domain consisting of an oligosaccharide binding fold made up of six β-sheets (β_1_-β_5b_) and an α-helix (α_3_), and a C-terminal β-grasp motif consisting of a large antiparallel β-sheet (β_6_, β_7a_, β_7b_, β_9_, β_10_, and β_12_) packed against three α-helices (α_2_, α_4_ and α_5_), and a two-stranded β-sheet (β_8_, β_11_) (Fig. 1A and Supplementary Fig. S2). The substitution in SEA, phenylalanine 47 to alanine, is not visible in the structure due to flexibility in that region. Furthermore, this change is distant from the TCR binding site and is therefore unlikely to directly affect binding of TCR. To compare the TCR specificity between SEA and the superantigen SEE, we also crystallized the SEE-TCR complex and determined the structure to 2.4 Å resolution (Supplementary Fig. S1 and Supplementary Fig. S2). This structure has previously been determined and published; however, the data set used in this analysis exhibits improved refinement statistics and resolution (Table 1)^22^. An overlay of the two structures show only small variations between them (Supplementary Fig. S1). The structure was refined to a R_work_ of 21.19% and R_free_ of 24.16%, and 99.7% of residues in the preferred or allowed region of the Ramachandran plot, and 0.3% in the generously allowed region (Table 1). Here, Asn52 in TRBV7-9 (that was in the disallowed region in the SEA-TCR structure) is in the generously allowed region, but adopts a similar conformation as in the SEA^F47A^-TCR structure. Compared to the superantigens SEB, SEC3, and SEH, which are the closest relatives that have been determined in complex with TCRs, the overall three-dimensional folds of SEA and SEE are similar (Supplementary Fig. S4), with pairwise RMSD values between Cα atoms listed in Supplementary Table S3. From this, it is evident that SEA and SEE are structurally very similar, with an RMSD of 0.789 Å, whereas SEH is not as similar, with an RMSD of 1.31 Å to SEA and 1.43 Å to SEE. Lastly, the SAgs SEB and SEC3 are more distantly related, with pairwise RMSDs to SEA and SEE between 1.59–1.68 Å (Supplementary Table S3).

### The Interfaces Between TCR and Staphylococcal Enterotoxins A and E

In both the SEA and the SEE structures, the SAgs utilize a shallow groove between the N- and C-terminal domains to engage with the TRBV domain of TCR (Fig. 1A and Supplementary Fig. S1). The buried surface area between the SAg and TRBV is 1817 Å^2^ for SEA-TCR and 1887 Å^2^ for SEE-TCR. These areas are relatively large, but the affinities are still in the micromolar range, 0.7 μM for SEA and 6 μM for SEE (Supplementary Table S4)^26^. This is likely due to the absence of salt bridges in the interfaces. For SEE-TCR, the even lower affinity as compared to SEA is likely due to lower solvation energy gain upon complex formation, −2.6 kcal/mol compared to −7.9 kcal/mol for SEA as calculated by the PISAserver^27^, also a less hydrophobic interface than SEA and therefore lower gain from the hydrophobic effect, probably contribute to a lower affinity. The TRBV domain of TCR mainly uses the CDR2 loop (37% and 41% of the buried surface area in TCR for the SEA-TCR and SEE-TCR complexes, respectively), the FR3 region (20% and 23%), and HV4 (23% and 19%), with smaller contributions from CDR1 (10% and 7.8%) as well as FR4 (10% and 7.8%). There are nine hydrogen bonds in the SEA-TCR complex and 16 in the SEE-TCR complex (Table 2 and Supplementary Table S1). Of these, six hydrogen bonds are conserved between both complexes. There are four regions of SEA involved in binding TCR: the α_2_-helix (residues 20, 21, 24, 25, 27, and 32–34), the hydrophobic patch consisting of the β_2_-β_3_ and β_4_-β_5a_ loops (residues 62–64, and 91–94), the α_4_-β_9_ loop (residues 174 and 175), and the N-terminal side of the α_5_-helix (residues 205 and 206) (Fig. 1B–E and Supplementary Fig. S3). In the SEA-TCR complex, 16 residues from SEA and 19 residues from TCR are involved in intermolecular van der Waals contacts (distance between residues less than 4 Å) (Supplementary Table S2). In comparison, 19 residues from SEE and 23 residues from TCR contribute to the buried surface area in SEE-TCR, reflecting its larger buried surface area (Supplementary Table S2 and Supplementary Fig. S3).

The structure presented here of SEE-TCR, and the previously determined structure of SEE-TCR are very similar (Supplementary Fig. S1)^22^, with RMSD for Cα atoms in TCRα, TCRβ, and SEE of 0.97 Å, 0.67 Å, and 0.62 Å, respectively. Both structures exhibit the same number of hydrogen bonds across the interface, with the same residues participating in hydrogen bonding, apart from Ser24 in SEE and Asp64 in TRBV, although some bonds are made between different atoms (Supplementary Fig. S1). Together, these structures illustrate that a certain degree of flexibility is allowed in the SEE-TCR interface.

### Buried Residues in the Superantigen-TCR Interfaces

**Figure 2:**
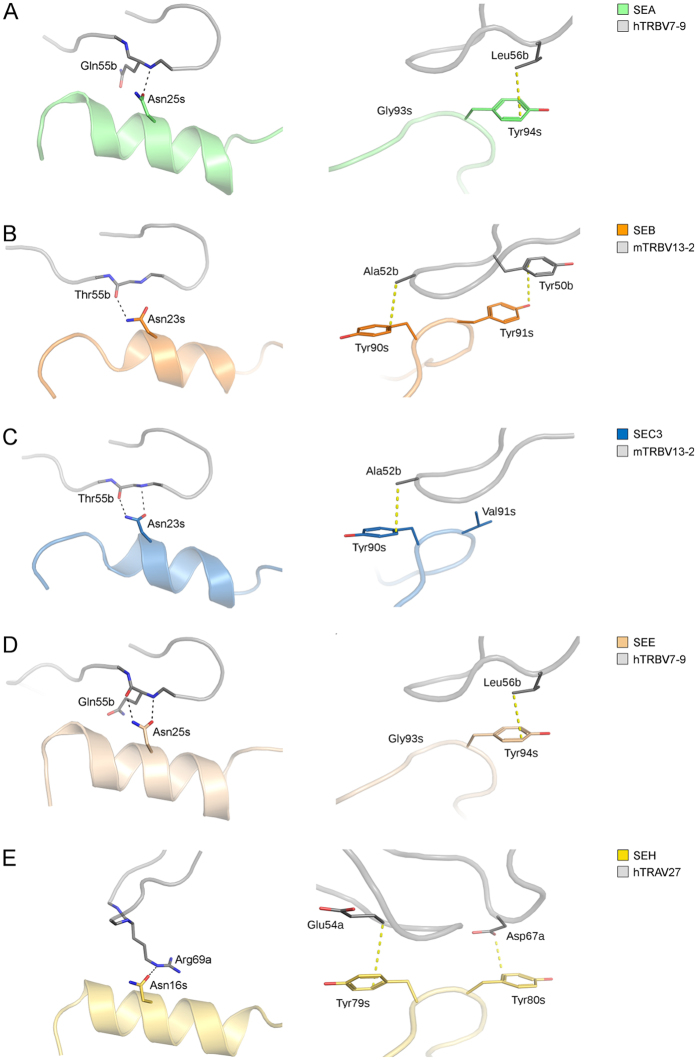
Close-up view of the conserved hot-spot regions. The hydrogen bonding asparagines (Asn25, SEA numbering) in the α_2_-helix of the superantigens and contacts involving π electrons of the phenyl rings of the aromatic pair. The superantigens are colored (**A**) SEA, (**B**) SEB, (**C**) SEC3, (**D**) SEE, (**E**) SEH, and the TCRs are shown in grey. Residues are designated as “s” for SAg, “a” for TRAV and “b” for TRBV. The hydrogen bonds and the XH/π or CH/π interactions are marked as black and yellow dotted lines, respectively.

To analyse the interfaces between the SEA and SEE-TCR complexes and to evaluate the contribution of specific residues, the solvent accessible surface area (ASA) for residues in the interface that becomes buried upon complex formation, was calculated using the PDBePISA server^27^. Residues with buried surface area (BSA) of ≥80 Å^2^ as well as residues for which the ASA becomes buried to 85% or more (%BSA), upon complex formation, are listed in Table 3. For SEA^F47A^-hTRBV7-9 there are five amino acids fulfilling either of these two conditions, Asn25, Tyr32, Trp63, Tyr94 and Phe175. Asn25 and Tyr32 are situated on the α_2_-helix, Trp63 is on the β_2_-β_3_ loop, Tyr94 on the β_4_-β_5a_ loop and Phe175 is situated on the α_4_-β_9_ loop (Fig. 1). In the SEE-hTRBV7-9 complex, corresponding amino acids for four of these residues were also found to have a large BSA and/or to become occluded from the solvent of more than 85% (Table 3). In addition to these four, Asn21 and Gln28, both situated in the α_2_-helix region, substantially contribute to the interface as they become almost entirely buried with % BSA of 99% and 97%, respectively. The previously determined structures of SEB, SEC3 and SEH in complex with TCR are highly similar to the structures between SEA-TCR and SEE-TCR presented here (Supplementary Fig. S4 and Supplementary Table S3)^14^,^16^,^28^. To investigate the fine differences between the SAgs, these structures were also submitted to the PISA server using their respective co-crystal structures (PDB ID: 1SBB, 3BYT and 2XNA), and the resulting values of their interface residue BSA and % BSA are presented in Table 3. In the SEB/SEC3-TCR structures, three residues stand out as gaining a high % BSA upon complex formation – two residues in the α_2_-helix region (with position 20 and 23) and a phenylalanine at position 177 (SEB numbering) situated on α_4_-β_9_ loop (Table 3). Moreover, Tyr91 in SEB has a BSA of 88 Å^2^, hence this residue is also likely to be important for the SEB-TCR interface. For the SEH-hTRAV27 complex, three residues stand out as important for the complex assembly - Asn16 (corresponds to Asn25 in SEA) and Gly19 located on the α_2_-helix of SEH^16^, which both become largely non-accessible to the solvent, and the Tyr79 residue with a BSA of 83 Å^2^. Taken together, these results suggests that the α_2_-helix and in particular the conserved Asn25 (SEA-numbering) contribute to complex formation of all SAg-TCR complexes studied. This conserved asparagine participates in hydrogen bonding in all complexes, which even further supports an important role for this residue when binding TCR (Fig. 2). In addition, specific aromatic side chains are likely to be of importance for the complex formation, but these are not always conserved among the complexes studied (Fig. 2).

### Computational Alanine-scanning Mutagenesis

**Figure 3:**
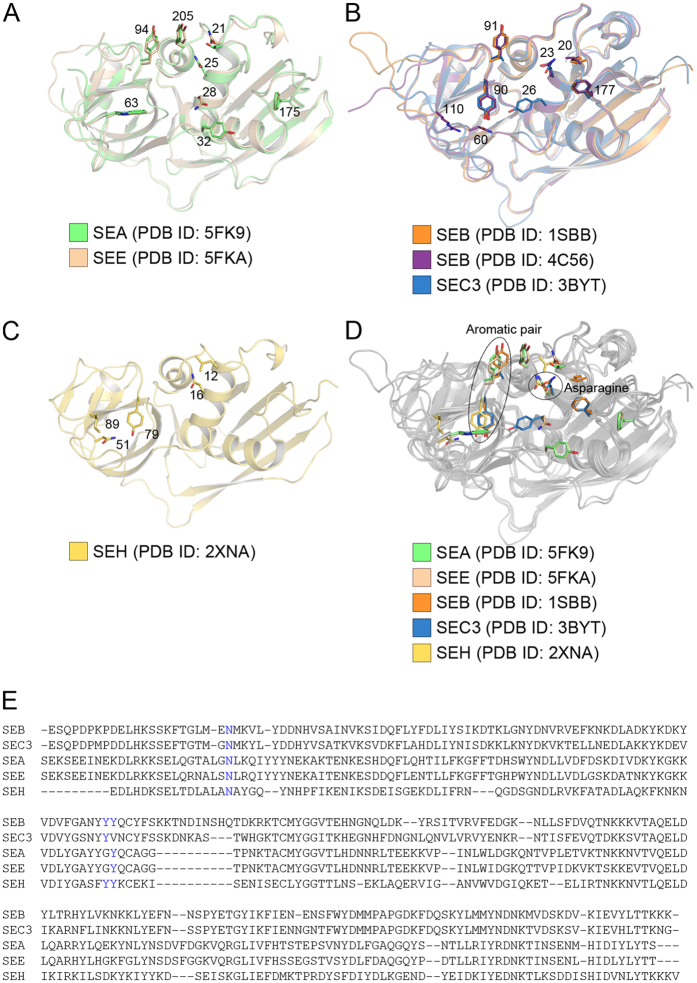
Ribbon representations of the superantigens investigated by *in silico* alanine-scanning mutagenesis. A structural overlay of (**A**) SEA and SEE, (**B**) SEB and SEC3, (**C**) SEH, (**D**) SEA, SEB, SEC3, SEE and SEH. Hot-spot residues for which alanine substitutions have destabilizing effects on ΔΔG_complex_ of more than, or equal to 1 kcal/mol, are shown as sticks. (**E**) Sequence alignment of the five superantigens (SEA, SEB, SEC3, SEE and SEH).

Computational alanine scanning mutagenesis was performed using ROBETTA, as described by Kortemme & Baker (ref. 29), to estimate a ΔΔG value of critical residues for complex formation^29^. ROBETTA has been proven to be an outstanding prediction tool to evaluate protein-protein interactions with approx. 80% correct predictions^30^. Each residue in the interface was substituted individually to an alanine, and the predicted effect of the substitution on the binding energy of the complex was calculated as ΔΔG_complex_ (Table 4 and Supplementary Table S5). Hot-spot residues are defined as those alanine substitutions on the SAg having a destabilizing effect on the ΔΔG_complex_ of more than or equal to 1 kcal/mol^30^. As seen in Table 4, most (six out of seven) of the hot-spot residues are common between SEA and SEE (Fig. 3A, Table 4). Clearly, Trp63 in both SAgs is most affected by alanine substitutions. Next, the structures of SEB and SEC3 were subjected to the same alanine scanning simulation protocol. Since these structures have been determined in association with the same mouse TRBV (mTRBV13-2), it allows us to investigate how a distinct pair of superantigens interacts with another TRBV (Fig. 3B, Table 4). As seen for SEA and SEE, most (four out of five) of the amino acids that are affected by alanine substitution in SEB and SEC3 are conserved amino acids. Phe177 in SEB and the corresponding Phe174 in SEC3, have the most pronounced effects (Table 4). SEB has also been crystallized with a human TCR (hTRBV19)^31^. To compare how SEB recognizes different TRBVs, the SEB-hTRBV19 (PDB ID: 4C56) interface was also subjected to alanine scanning mutagenesis. All residues that significantly contribute to the complex formation of SEB-mTRBV13-2 also contribute to the SEB-hTRBV19 interface (Table 4). However, there are three additional residues with a ΔΔG_complex_ value of more than 1 kcal/mol in the SEB-hTRBV19 complex, suggesting that the affinity is higher between hTRBV19 and SEB than for SEB-mTRBV13-2 complex (Table 4). This inference is corroborated by previously published data, which reports the affinity for hTRBV19 to be approximately twice as high as for mTRBV13-2 (Supplementary Table S4)^32^,^33^. Lastly, we performed alanine scanning mutagenesis on the SEH-TCR complex. SEH is the only staphylococcal superantigen that has been demonstrated to bind the human TRAV domain of TCR (hTRAV27)^11^,^16^. Interestingly, the amino acid with largest ΔΔG_complex_ value (Asn16, SEH-numbering) have corresponding residues in SEA, SEE, SEB and SEC_3_, which appear to be hot-spot residues for those complexes as well (Fig. 3D, Table 4). These residues are sequentially conserved among all five SAgs (Fig. 3E). Moreover, the amino acid with the second largest ΔΔG_complex_ value for SEH-hTRAV27 (Tyr79), is well conserved among the other SAgs as well (Fig. 3). When examining these two common areas in the crystal structure of each SAg-TCR complex, it is evident that the Asn25 (SEA numbering) forms hydrogen bonds to the TCR molecule, while the tyrosine residues can potentially interact with their respective TCR due to their aromatic properties (Fig. 2).

## Discussion

To identify which residues of SEA that are important for TRBV specificity, and which ones serve as common anchor points for superantigens in general, we have determined the three-dimensional structure of SEA in complex with a human T cell receptor, performed computational alanine scanning mutagenesis, and compared to previously structurally investigated SAgs, *i.e.* SEB, SEC3, SEE and SEH. While SEE is crystallized with a TCR containing hTRBV7-9 (as SEA), the structures for SEB and SEC3 are complexed with murine TRBV13-2^14^,^15^. Hence, we have two pairs of superantigens (SEA/SEE and SEB/SEC3) crystallized with the same TRBV domain. This allowed us to directly investigate the conserved mechanisms that permit different superantigens to recognize the same TCR. Furthermore, we also assessed the TRAV binding superantigen SEH, which has been crystallized with a TCR containing human TRAV27^16^. This complex was also subjected to computer based alanine scanning mutagenesis to probe for common recognition sites between these two discrete TCR domains. Finally, we have performed multiple sequence/structural alignment among the complexes to identify motifs that can be targeted when designing superantigen blockers.

The overall structures of SEA in complex with hTRBV7-9 and SEE in complex with hTRBV7-9 are very similar. Out of the nine hydrogen bonds present in the SEA-hTRBV7-9 structure, six are conserved in the SEE-hTRBV7-9 structure. In addition, the residues that become almost entirely buried upon SAg-TCR complex formation in SEA (≥85% BSA or ≥80 Å^2^ BSA as calculated by PISA) are also highly buried within the interface in SEE, indicating further similarities in complex formation for the two SAgs. Also, the six residues identified by *in silico* alanine scanning that affect the complex formations with ΔΔG_(complex)_ ≥ 1 kcal/mol are the same in SEA and SEE. Among these, four residues form similar hydrogen bonds in both SAgs. The fifth conserved hydrogen bond, from Asn25, only shows up as a significant amino acid in the alanine scan of SEA but not for SEE. The sixth hydrogen bond is from the backbone of Val174 (Ser174 in SEE), and thus is not affected by an alanine substitution. Interestingly, the results are very similar within the SEB/SEC3 pair as well; four of the five residues identified by *in silico* alanine scanning for mTRBV13-2 binding, are structurally and sequentially conserved between SEB and SEC3, and interfacial residues with a large buried surface area are conserved between SEB and SEC3. This suggests that superantigens utilize the same residues to bind a particular TRBV, which is supported by previous studies where a single antagonist could *in vitro* block the activity of both SEC3 and SEB to a particular TRBV^34^.

**Figure 4:**
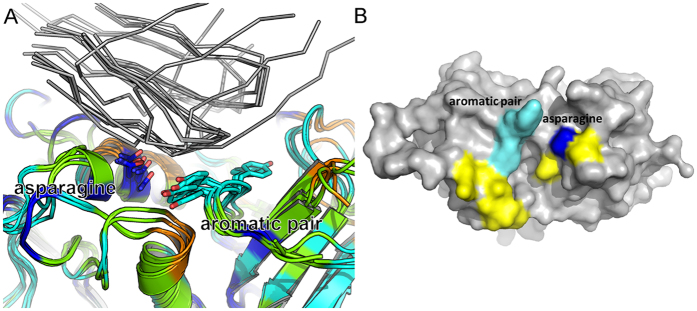
Superantigen superposition and conservation. (**A**) SAg superpositions are shown colored by sequence conservation (blue is completely conserved, red is completely divergent). Conserved regions of interacting TCR elements are each shown as grey C_α_ traces. (**B**) Superantigen residues that serve as anchor-points for SAg-TCR complexes; the conserved asparagine (blue) and the aromatic pair (cyan), and residues suggested to be important for TRBV discrimination are colored in yellow.

SEB has also been crystallized with hTRBV19^31^. To determine whether SEB utilizes conserved structural motifs for different TRBVs, this structure was also subjected to *in silico* alanine scanning mutagenesis. All SEB residues that are hot-spots in the SEB-mTRBV13-2 complex were also identified as hot-spots in the SEB-hTRBV19 complex (Fig. 3B, Table 4). In addition, there are three extra amino acids (Leu58, Asn60 and Arg110) contributing to the SEB-hTRBV19 interface, and these are likely responsible for the higher estimated affinity of this complex (Supplementary Table S4)^31^,^32^. These three residues might be important for conferring TRBV specificity to SEB (Fig. 4B). This observation is supported by previous data, where substitution of the residue at position 60 (in the β_2_-β_3_ loop) affected the TRBV specificity^35^. In the corresponding β_2_-β_3_ loop on SEA, the residue at position 60 was also proposed to be important for epitope specificity (Fig. 4B)^36^. In both pairs of SAgs we analysed (SEA/SEE and SEB/SEC3), there is one amino acid difference within each pair. In both cases, this difference is within, or near the α_2_-helix of the superantigen (Figs 3A,B and 4B, Table 4), possibly contributing to TRBV selectivity. This hypothesis is supported by previously published data, where this region has been shown to be essential for determining the TRBV specificity of SEA and SEE^37^.

When evaluating the common hot-spot residues for all five superantigens (SEA, SEB, SEC3, SEE and SEH) two distinct features were identified that were common to four out of the five superantigens. The first hot-spot is Asn25 (SEA numbering), which is within the α_2_-helix and contributes significantly to the complex formation as well as the binding energy. This asparagine is conserved in all five SAg structures we assessed, and it participates in intermolecular hydrogen bonds with the respective TRBV or TRAV binding partner (Fig. 2)^14^,^15^,^16^. However, in SEE it has a ΔΔG_complex_ value of 0.93 kcal/mol in the *in silico* analyses, hence just below the border value for qualification as a hot-spot residue. Still, it forms two hydrogen bonds to the TCR molecule as seen in the X-ray structure and it is highly buried within the interface of the SEE-TCR complex, which suggests it to be a crucial residue for complex formation in SEE as well. The importance of this residue to the SAg-TCR interactions is also supported by previously published data; the corresponding asparagine-to-alanine substitution in SEB has been shown to be important for binding to numerous TRBVs^38^. Moreover, Leder and co-workers evaluated the most important residues for the mitogenic potency of SEC3, revealing that substitution of Asn23 to alanine (corresponding to Asn25 in SEA) effectively extinguished mitogenic activity^39^.

The second hot-spot region, which is common to all five complexes, is the aromatic pair of residues Tyr90 and Tyr91 (SEB numbering) (Fig. 3B). Two tyrosines, as observed in SEB, do not seem to be an absolute requirement, but one of these tyrosines is always present (Fig. 2). Residues with aromatic side chains are known to contribute to a protein structure via a variety of contacts involving the π electrons of the phenyl ring and have been described to serve as hydrogen bond acceptors^40^. Such XH/π interactions have energies of 1–4 kcal/mol^41^. Interestingly, even the weaker CH/ π interactions have been suggested to be important in protein structures^37^. Indeed, in all five superantigens studied, one or two tyrosines of the aromatic pair contribute to the complex formation, as they are located in the interface with a TCR molecule and can interact with the corresponding residues of the TCR via π electrons (Fig. 2). Moreover, *in silico* alanine scanning also consistently identified these residues as significant contributors to binding energy (Fig. 3B, Table 4). In SEC3, substitution of Tyr90 to alanine has previously been shown to result in nearly abrogated TCR binding and severely reduced mitogenic activity^39^. Furthermore, substitution of Tyr91 to valine in SEB has also been shown to result in a decreased mitogenic activity, and substitution of the residue corresponding to Tyr91 in SEC3 (Val91) to alanine has been demonstrated to be a contributing factor in the decreased mitogenic activity of SEC3^39^. Along these lines, the corresponding residue in SEA (Tyr94) has been shown to be important for T cell activation^20^. In the case of SEH, both tyrosines are conserved, but only one (Tyr79, corresponding to Tyr90 in SEB) contributes significantly to complex formation according to the *in silico* alanine scan (Table 4).

All SAgs in our analysis (SEA, SEB, SEC3, SEE, and SEH) interact with common TCR loops via a conserved binding pocket. Multiple sequence/structure alignment of superantigen structures using SALIGN revealed significant sequence divergence at the protein surface (Fig. 4A). Interestingly, there is no significant increase in conservation at the common TCR interfaces in comparison to the rest of the surface. The notable exceptions are the asparagines (residue 25 by SEA numbering), which are strictly conserved and the aromatic pair of tyrosine residues that also exhibit a high degree of sequence/structure conservation (Figs 3E and 4A). Despite the sequence variability, the overall shapes of SAgs are highly similar, as is necessary for the conserved interaction with TCRs (Supplementary Fig. S4). Taken together, these data indicate there are two important regions conserved amongst these SAgs that are crucial for TCR binding to take place: the conserved asparagine on the α_2_-helix, and the aromatic pair (Fig. 4B). Other surrounding residues are perhaps more important for TCR discrimination (Fig. 4B). A significant challenge remaining is the design of neutralizing SAg binders with broad spectrum activity. *In silico* alanine-scanning and structural analysis both suggest that there are no conserved TCR residues that contribute significantly to complex formation with SAgs (Supplementary Table S5). Furthermore, mimicking the TCR would be difficult since a part of the specificity lies in the conformation of the C” strand, and does not seem to be dependent on side-chain interactions^33^. Thus, it would be possible to make TCR mimicking agonists within SAg groups, but it would be difficult between groups. Furthermore, the binding mode of TRAV27 to SEH is very different as the TRAV domain is rotated with 180 degrees as compared to the TRBV binding mode to the other SAgs^16^. Hence, methods for protein interface design, which utilize a set of pre-defined hot-spot residues^42^ are not directly applicable. We suggest that efforts to engineer binders aimed at broad neutralization of SAgs should instead focus on two elements; first, interaction with the highly conserved asparagine on the α_2_-helix; and second, shape complementarity for the remainder of the interface, in particular occluding the aromatic pair.

## Methods

### Protein Expression and Purification

The T cell receptor α- and β-chains (α-chain TRAC*01, TRAJ40*01,TRAV22*01 and β-chain TRBC2*01, TRBJ2-3*01, TRBV7-9*01) were expressed and refolded together as described previously^24^,^43^, apart from minor changes^22^. The α- and β-chains of TCR were expressed separately in *Escherichia coli* BL21(DE3)Star cells (Invitrogen), by induction with 0.5 mM IPTG. Each cell pellet was resuspended in 100 ml homogenization buffer (50 mM Tris-HCl pH 8.0, 750 mM sucrose, 1 mM EDTA, 10 mM DTT), followed by addition of 250 ml lysis buffer 20 mM Tris-HCl pH 8.0, 100 mM NaCl, 1% (w/v) deoxycholate, 1% (v/v) Triton X-100, 10 mM DTT, 0.3 mg/ml lysozyme), supplemented with 0.5 mM MgCl_2_ and 20 μg/ml DNAse I. The lysate was subjected to one round of freeze thawing and inclusion bodies were collected by centrifugation. Inclusion bodies were then washed in buffer A (50 mM Tris-HCl pH 8.0, 100 mM NaCl, 1 mM EDTA, 0.5% (v/v) Triton X-100, 10 mM DTT) and collected by centrifugation. This was repeated twice and followed by one round of washing in buffer A without NaCl and Triton X-100. Inclusion bodies were then solubilized in 50 mM Tris-HCl pH 8.0, 6 M guanidine, 100 mM NaCl, 10 mM EDTA, 5 mM DTT. For refolding, α-chain at 0.2 mg/ml and β-chain at 0.15 mg/ml were refolded together in 100 mM Tris-HCl pH 8.0, 5 M urea, 400 mM L-arginine, 0.83 mg/l cysteamine hydrochloride, 0.73 mg/l cystamine dihydrochloride and dialyzed for 48 h, first against Milli-Q and then against 10 mM Tris-HCl pH 8.0. The refolded protein was filtered through a 0.2 μm filter and purified with ion exchange chromatography using a Resource Q 6 ml column (GE Healthcare) in 20 mM Tris-HCl pH 8.0 with a gradient of 0–400 mM NaCl, followed by size exclusion chromatography in TBS buffer, using a Superdex 200 10/300 GL column (GE Healthcare).

Purified SEA^F47A^ mutant and SEE were provided by Active Biotech Research AB, which had been purified according to published protocols^44^.

### Crystallization and Data Collection

The SEA^F47A^-TCR complex was crystallized in 24% PEG 2000 MME, 0.1 M NH_4_Cl pH 8.5, and 0.05 M NaCl at a SEA^F47A^:TCR ratio of 1:1 and a total protein concentration of 3.6 mg/ml, using vapour diffusion at 20 °C and formed during 6–8 weeks. Crystals were soaked in reservoir solution containing 20% glycerol as cryo protectant and data was collected at the ESRF beamline ID23-2 at a wavelength of 0.87260 Å and 100 K, with an oscillation range of 0.2°. SEE-TCR crystals formed over 4–6 weeks at 20 °C in 25% PEG 3350, 0.1 M Glycine pH 9.0, and 0.1 M NaCl at a total protein concentration of 6.5 mg/ml at a SEE:TCR ratio of 1:1. Prior to mounting crystals in a cryo loop, reservoir solution containing 20% glycerol was added to the drop. The SEE-TCR data was collected at the ESRF beamline ID14-4 at λ 0.97625 Å and 100 K, with an oscillation of 0.5°.

### Structure Determination and Refinement

Diffraction data of both structures were integrated and merged with XDS^45^ and aimless^46^,^47^, within autoPROC^48^, with 5% of the data chosen as a subset for cross-validation. The structures were solved with molecular replacement using Phaser^49^. The TCR structure determined previously (PDB ID: 4UDT)^22^ was used as a search model for the TCR, whereas SEA (PDB ID: 1ESF)^50^ and SEE in the previously determined SEE-TCR structure (PDB ID: 4UDU)^22^ were used as models for the superantigens. The structures were then refined in refmac5^51^ within the CCP4 suite^17^ and autoBUSTER^52^, with manual model building rounds in Coot^53^. Composite omit maps were calculated with CNS^54^ for both structures, omitting 5% of the models. Ramachandran statistics for the SEA^F47A^-TCR and SEE-TCR structures were calculated in SFCHECK^55^ (Table 1). All figures depicting structures were produced using PyMOL (Schrödinger LLC). The % BSA was calculated as a fraction of BSA/ASA (percentage accessible surface area buried upon complex formation) using the PDBePISA server^27^, and the multiple structure/sequence alignments were performed using SALIGN^56^ and Clustal Omega^57^. RMSD values between structures were calculated using superpose^58^.

### Computational Alanine-scanning Mutagenesis

Computational alanine scanning mutagenesis was performed on five different protein complexes, SEA^F47A^-TCR (PDB: 5FK9), SEE-TCR (PDB: 5FKA), SEB-TCR (PDB: 4C56), SEB-TRBV (PDB: 1SBB), SEC3-TRBV (PDB: 3BYT), using ROBETTA ( http://robetta.bakerlab.org/alascansubmit.jsp). The ROBETTA protocol has been previously described by Kortemme & Baker^29^. Briefly, two partners in each structure (the superantigen and the TRBV) were selected and all residues in the interface between the two partners were individually substituted to an alanine and the predicted effect on the binding energy of the protein-protein complexes were computed, to find the hot-spots for complex formation. Hot-spot residues were defined as those for which alanine substitutions have predicted destabilizing effects on ΔΔG_complex_ of more than or equal to Rosetta energy units (approximating 1 kcal/mol).

## Additional Information

**Accession codes:** The atomic coordinates and structure factors have been deposited in the PDB under accession codes PDB: 5FK9 and 5FKA. 

**How to cite this article**: Rödström, K. E. J. *et al*. Two common structural motifs for TCR recognition by staphylococcal enterotoxins. *Sci. Rep.* **6**, 25796; doi: 10.1038/srep25796 (2016).

## Supplementary Material

Supplementary Information

## Figures and Tables

**Table 1:** Data collection and refinement statistics.

	**SEA**^**F47A**^**-TCR**	**SEE-TCR**
**Data collection**		
Space group	P2_1_2_1_2	P2_1_2_1_2
Cell dimensions		
*a*, *b*, *c* (Å)	114.3, 150.4, 39.69	113.3, 150.1, 39.16
*α, β, γ* (°)	90, 90, 90	90, 90, 90
Resolution (Å)	45.9–3.09 (3.26−3.09)	45.8−2.40 (2.53−2.40)
No. reflections/unique	36,678/12,028	176,772/26,772
*R*_merge_	0.124 (0.720)	0.104 (0.678)
*I*/*σI*	14.4 (2.8)	16.3 (1.9)
Completeness (%)	92.4 (90.9)	98.8 (92.4)
Redundancy	3.0 (3.0)	6.6 (4.3)
**Refinement**		
Resolution (Å)	3.1	2.4
*R*_work_/*R*_free_	0.2571/0.2908	0.2119/0.2416
No. atoms		
Protein	4767	5102
Zinc	–	1
Water	4	94
B-factors		
Protein	57.91	38.72
Zinc	–	66.31
Water	14.15	30.58
R.m.s. deviations		
Bond lengths (Å)	0.007	0.007
Bond angles (°)	0.880	1.020
Ramachandran statistics		
Favored (%)	87.2	91.6
Allowed (%)	12.1	8.1
Generously allowed (%)	0.5	0.3
Disallowed (%)	0.2	0

Data were collected from single crystals. Values in parentheses are for the highest resolution shell.

**Table 2:** Intermolecular hydrogen bonds in the SEA^F47A^-TCR structure.

**Residue SEA**	**Atom**	**Residue TRBV**	**Atom**	**Distance [Å]**
Thr21	Oγ1	Gln55	O	2.7
Asn25	Oδ1	Gln55	N	2.9
Tyr32	Oη	Glu69	Oε1	2.7
Trp63	Nε1	Ser28	O	2.9
Trp63	Nε1	His30	O	2.8
Gly93	N	Glu53	Oε1	2.8
Tyr94	N	Glu53	Oε1	2.9
Val174	O	Gln81	Nε2	2.8
Tyr205	Oη	Leu56	O	3.0

**Table 3:** Analysis of buried surface areas.

SEA-hTRBV7-9 (B:C)*Residue BSA			SEE-hTRBV7-9 (B:C)*Residue BSA			SEB-mTRBV13-2 (A:B)*Residue BSA			SEC3-mTRBV13-2 (A:B)*Residue BSA			SEH-hTRAV27 (A:C)*Residue BSA		
	*%*	*Å*^*2*^		*%*	*Å*^*2*^		*%*	*Å*^*2*^		*%*	*Å*^*2*^		*%*	*Å*^*2*^
Asn25	96	35	Asn21	99	68	Leu20	94	77	Thr20	93	47	Asn16	85	25
Tyr32	62	85	Asn25	97	30	Asn23	93	49	Asn23	89	55	Gly19	95	22
Trp63	92	163	Gln28	97	62	Tyr91	48	88	Phe174	85	118	Tyr79	71	83
Tyr94	63	93	Trp63	90	153	Phe177	93	99						
Phe175	76	113	Tyr94	73	95									
			Phe175	75	126									
			Pro206	89	14									

Residues in the SAgs that have a buried surface area of ≥85% and/or of ≥80 Å^2^ upon complex formation according to the PDBePISA server. *Chain ID of the structure models that were used for the interface analysis. The conserved asparagine and the hydrophobic pair are underlined.

**Table 4:** Residues in the superantigens affected by *in silico* alanine-scanning mutagenesis.

Residue	ΔΔG_complex_	Residue	ΔΔG_complex_
**SEA-hTRBV7-9**		**SEE-hTRBV7-9**	
Thr21	1.34	Asn21	2.10
Asn25	1.07	Gln28	1.43
Tyr32	1.40	Tyr32	2.17
Trp63	4.84	Trp63	5.24
Tyr94	1.72	Tyr94	1.91
Phe175	3.03	Phe175	3.54
Tyr205	1.37	Tyr205	1.28
**SEB-mTRBV13-2**		**SEC3-mTRBV13-2**	
Leu20	1.76	Asn23	0.96
Asn23	1.50	Tyr26	1.01
Tyr90	1.27	Tyr90	1.43
Tyr91	1.68	Val91	1.00
Phe177	3.24	Phe174	2.84
**SEB-hTRBV19**		**SEH-hTRAV27**	
Leu 20	2.02	Leu12	1.46
Asn23	2.58	Asn16	4.09
Leu58	0.99	Asn51	1.34
Asn60	1.79	Tyr79	2.97
Tyr90	1.52	Ile89	1.02
Tyr91	1.85		
Arg110	1.44		
Phe177	3.31		

ΔΔG_complex_ values are shown in kcal/mol. The conserved asparagine and residues in the hydrophobic pair are underlined.
