# Supplementary material for: Two common structural motifs for TCR recognition by staphylococcal enterotoxins
Source: Sci Rep. 2016 May 16;6:25796. doi: 10.1038/srep25796 (PMC4867771; doi:10.1038/srep25796)
Supplement: Supplementary Information [file srep25796-s1.pdf]

Supplementary Information

Two common structural motifs for TCR recognition by staphylococcal enterotoxins

Karin Erica Johanna Rödström[1], Paulina Regenthal[1], Christopher Bahl[2], Alex Ford[2], David Baker[2], Karin Lindkvist-Petersson[1]

1. Department of Experimental Medical Science, Lund University, BMC C13, 22 184, Lund, Sweden.
2. Department of Biochemistry, University of Washington, and Howard Hughes Medical Institute, Seattle, Washington 98195, USA.

Supplementary Figures

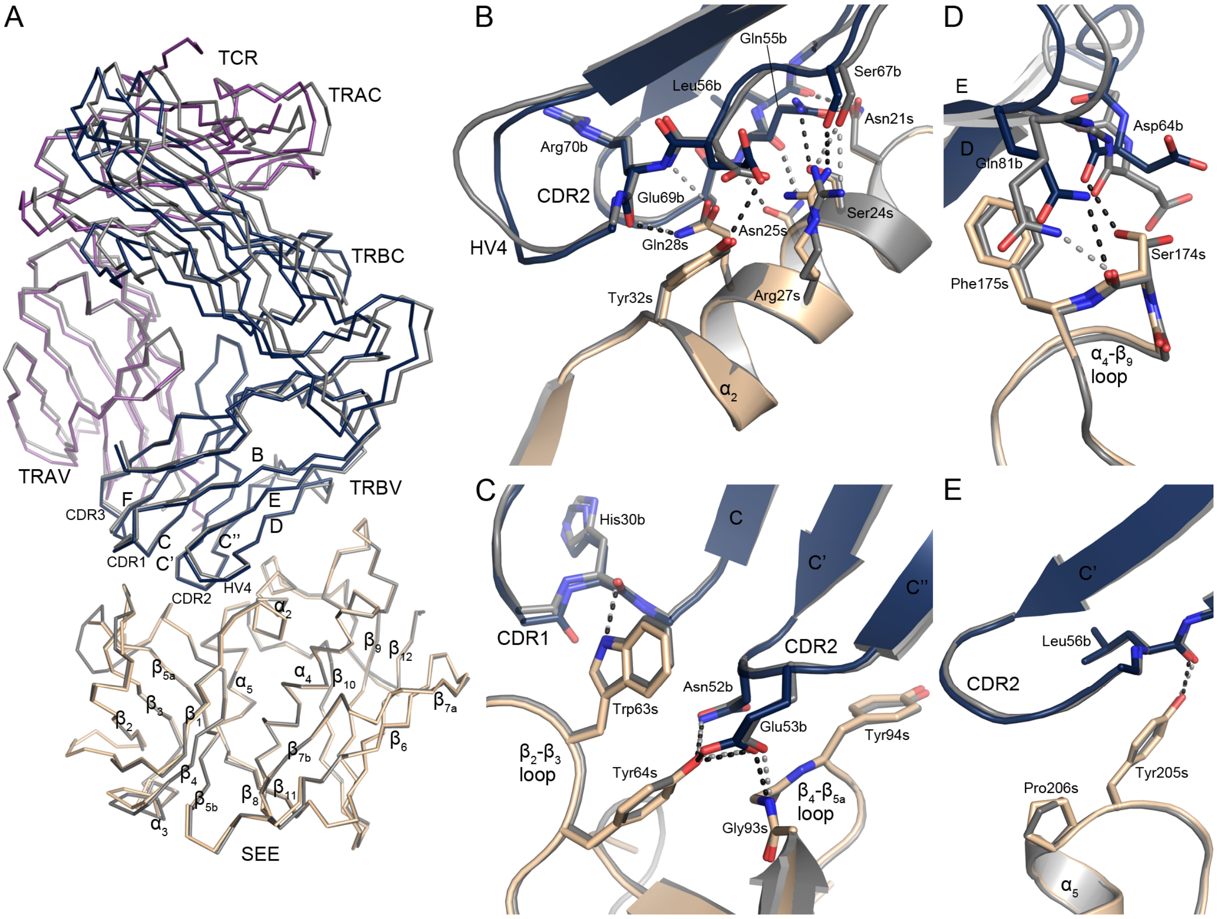

Figure S1. Structure of the SEE-TCR Complex. The present structure (PDB ID: 5FKA) (coloured) compared to the previously published SEE-TCR structure (PDB ID: 4UDU) (grey) (**A**) Overall structure of SEE-TCR, with SEE shown in beige, and TCR in purple and blue for the α- and β-chain, respectively. Close-up of the SEE α_2-helix (**B**) and contacting residues in TCR, the hydrophobic patch, consisting of the β_2-β_3 and β_4-β_{5a} loops (**C**), the α_4-β_9 loop (**D**), (**E**) and the upper part of the α_5-helix. Hydrogen bonds are marked as dotted lines and residues designated as "s" for SAg and "b" for TRBV7-9.

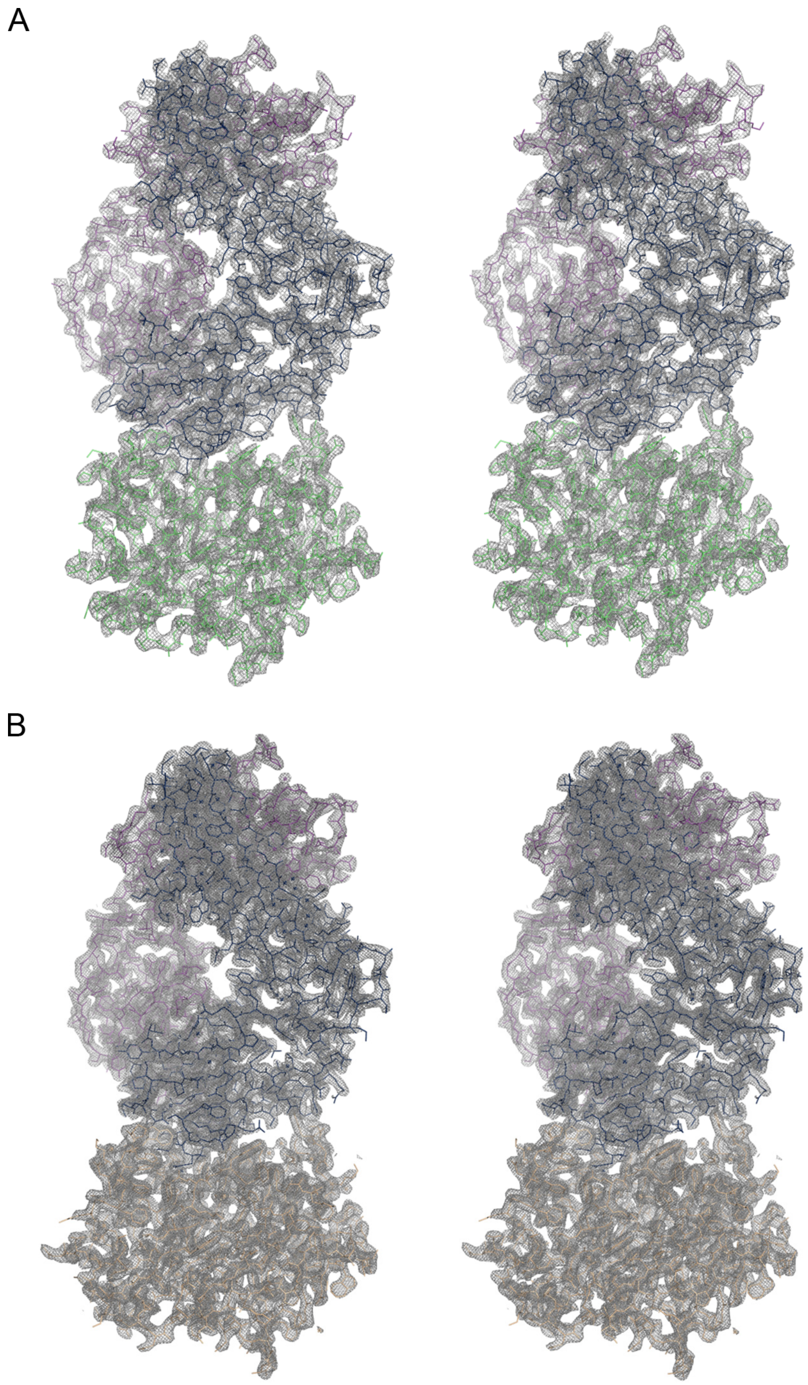

Figure S2. Stereo view of the 2F_o-F_c electron density maps of the structures. (**A**) SEA^{F47A}-TCR with SEA in green, TCRα in purple and TCRβ in blue. (**B**) SEE-TCR, with SEE in beige, TCRα in purple and TCRβ in blue. Structures are shown in stick representation, with the 2F_o-F_c maps contoured at 1.0σ.

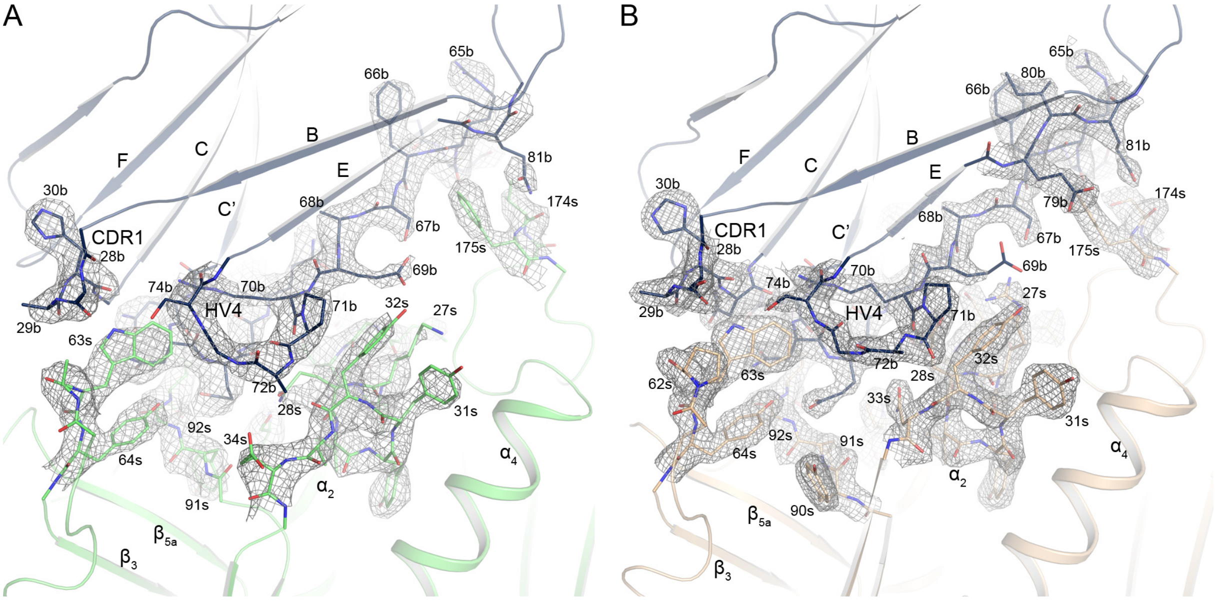

Figure S3. View of the SAg-TCR interfaces. The $2F_o$-F_c electron density maps contoured at 1.0σ are shown around interface residues in the (**A**) SEA^{F47A}-TCR and (**B**) SEE-TCR structures. Residues designated as "s" for SAg and "b" for TRBV7-9.

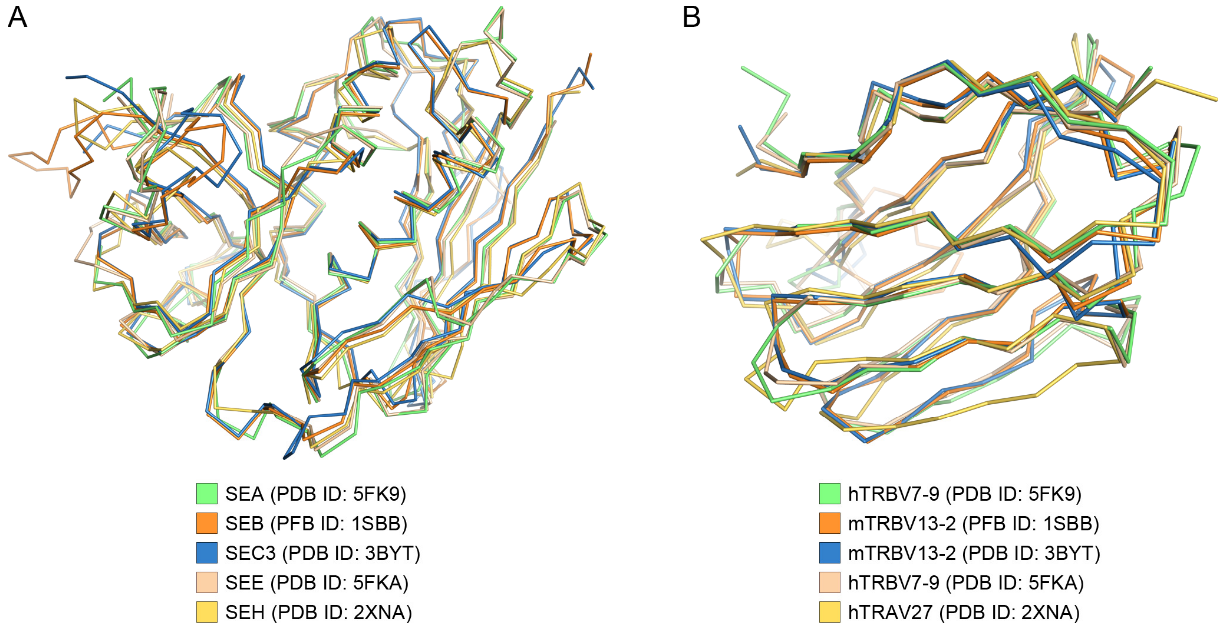

Figure S4. Alignment of the SAgs and TRBV/TRAVs investigated. (**A**) Superantigens SEB, SEC3, SEE, and SEH are aligned with respect to SEA (**B**) The hTRBV7-9, mTRBV 13-3 and hTRAV27 aligned with respect to hTRBV7-9 in the SEA^{F47A}-TCR structure. Alignments were performed using secondary structure matching and RMSD values are shown in Supplementary Table S3.

Supplementary Tables

Table S1. Intermolecular Hydrogen Bonds in the SEE-TCR Structure.

Residue SEE	Atom	Residue TRBV	Atom	Distance [Å]
Asn21	Oδ1	Gln55	Oε1	3.0
Asn21	Nδ2	Leu56	O	3.2
Ser24	Oγ	Gln55	Nε2	2.9
Asn25	Oδ1	Gln55	N	2.8
Asn25	Nδ2	Gln55	O	3.1
Arg27	Nη2	Ser67	Oγ	3.1
Gln28	Nε2	Arg70	O	3.0
Tyr32	Oη	Glu69	Oε2	2.9
Trp63	Nε1	His30	O	3.1
Tyr64	Oη	Asn52	Nδ2	2.9
Tyr64	Oη	Glu53	Oε1	3.0
Tyr64	Oη	Glu53	Oε2	2.9
Gly93	N	Glu53	Oε1	3.0
Ser174	O	Gln81	Nε2	3.1
Ser174	Oγ	Asp64	O	2.7
Tyr205	Oη	Leu56	O	2.6

Table S2. Intermolecular Van der Waals Contacts (distances less than 4 Å) in the SEA^{F47A}-TCR and SEE-TCR Complexes.

SEA	TCR	SEE	TCR
Thr21	Ser28	Arg20	Ser28
Asn25	Glu29	Asn21	Glu29
Lys27	His30	Ser24	His30
Gln28	Gln51	Asn25	Asn31
Tyr32	Asn52	Arg27	Gln51
Glu34	Glu53	Gln28	Asn52
Ser62	Ala54	Tyr32	Glu53
Trp63	Gln55	Asn33	Ala54
Tyr64	Leu56	Pro62	Gln55
Tyr92	Asp64	Trp63	Leu56
Gly93	Arg65	Tyr64	Glu57
Tyr94	Ser67	Tyr91	Lys58
Val174	Glu69	Tyr92	Asp64
Phe175	Arg70	Gly93	Arg65
Tyr205	Pro71	Tyr94	Phe66
Ser206	Lys72	Ser174	Ser67
	Gly73	Phe175	Ala68
	Ser74	Tyr205	Glu69
	Gln81	Pro206	Arg70
			Pro71
			Ser74
			Glu79
			Gln81

Table S3. Pairwise RMSD values (Å) between SAgs (chain IDs B or C) and variable TCR domains (chain IDs A or B), calculated using secondary structure matching.

SAg	SEA 5FK9 (C)	SEB 1SBB (B)	SEC3 3BYT (B)	SEE 5FKA (C)	SEH 2XNA (C)
SEA 5FK9 (C)	0	1.68	1.63	0.789	1.31
SEB 1SBB (B)		0	0.732	1.59	1.69
SEC3 3BYT (B)			0	1.60	1.66
SEE 5FKA (C)				0	1.43
SEH 2XNA (C)					0
**TRBV/TRAV**	**hTRBV7-9 5FK9 (B)**	**mTRBV13-2 1SBB (A)**	**mTRBV13-2 3BYT (A)**	**hTRBV7-9 5FKA (B)**	**hTRAV27 2XNA (B)**
hTRBV7-9 5FK9 (B)	0	1.41	1.46	0.700	1.43
mTRBV13-2 1SBB (A)		0	0.590	1.36	1.40
mTRBV13-2 3BYT (A)			0	1.44	1.46
hTRBV7-9 5FKA (B)				0	1.31
hTRAV27 2XNA (B)					0

Table S4. Equilibrium Dissociation Constants for the Studied Staphylococcal Enterotoxins to TCR.

SAg	TCR	K_d [µM]	Reference
SEA	hTRBV7-9	0.7	Hedlund et al. (2013)[1]
SEE	hTRBV7-9	6	Hedlund et al. (2013)[1]
SEB	mTRBV13-2	140	Malchiodi et al. (1995)[2]
SEB	hTRBV19	40-90	Rodstrom et al. (2014)[3]
SEC3	mTRBV13-2	3	Leder et al. (1998)[4]
SEH	hTRAV27	4	Pumphrey et al. (2007)[5]

Table S5. Residues in TCR Affected by *in Silico* Alanine-scanning Mutagenesis with $\Delta\Delta G_{complex}$ values shown in kcal/mol.

SEA-hTRBV7-9		SEE-hTRBV7-9	
**Residue**	**$\Delta\Delta G_{complex}$**	**Residue**	**$\Delta\Delta G_{complex}$**
Glu53	3.31	Asn52	1.07
Gln55	1.52	Glu53	1.83
Leu56	1.18	Leu56	1.12
Gln81	1.02	Glu57	1.30
		Gln81	1.45
**SEB-mTRBV13-2**		**SEC3-mTRBV13-2**	
**Residue**	**$\Delta\Delta G_{complex}$**	**Residue**	**$\Delta\Delta G_{complex}$**
-	-	Lys57	1.39
**SEB-hTRBV19**		**SEH-hTRAV27**	
**Residue**	**$\Delta\Delta G_{complex}$**	**Residue**	**$\Delta\Delta G_{complex}$**
Tyr49	1.11	Val28	1.07
Gln52	0.96	Arg69	4.62
Ile53	1.23		
Phe57	1.84		

Supplementary References

1 Hedlund, G. *et al.* The tumor targeted superantigen ABR-217620 selectively engages TRBV7-9 and exploits TCR-pMHC affinity mimicry in mediating T cell cytotoxicity. *PloS one* **8**, e79082, doi:10.1371/journal.pone.0079082 (2013).

2 Malchiodi, E. L. *et al.* Superantigen binding to a T cell receptor beta chain of known three-dimensional structure. *The Journal of experimental medicine* **182**, 1833-1845 (1995).

3 Rodstrom, K. E., Elbing, K. & Lindkvist-Petersson, K. Structure of the superantigen staphylococcal enterotoxin B in complex with TCR and peptide-MHC demonstrates absence of TCR-peptide contacts. *J Immunol* **193**, 1998-2004, doi:10.4049/jimmunol.1401268 (2014).

4 Leder, L. *et al.* A mutational analysis of the binding of staphylococcal enterotoxins B and C3 to the T cell receptor beta chain and major histocompatibility complex class II. *The Journal of experimental medicine* **187**, 823-833 (1998).

5 Pumphrey, N. *et al.* Cutting edge: Evidence of direct TCR alpha-chain interaction with superantigen. *J Immunol* **179**, 2700-2704, doi:179/5/2700 [pii] (2007).
